# Pleomorphic Giant Cell Carcinoma of the Prostate Treated With Lutetium‐177 PSMA

**DOI:** 10.1155/criu/1304419

**Published:** 2026-08-05

**Authors:** John Y. Kwon, John C. Cheville, Miguel Muniz-Rincon, Mohamed E. Ahmed, Eugene D. Kwon, Irbaz Riaz, Dan S. Childs, Geoffrey Johnson, Oliver Sartor, Lance C. Pagliaro, Jacob J. Orme

**Affiliations:** ^1^ Department of Internal Medicine, Mayo Clinic, Rochester, Minnesota, USA, mayo.edu; ^2^ Department of Laboratory Medicine and Pathology, Mayo Clinic, Rochester, Minnesota, USA, mayo.edu; ^3^ Department of Oncology, Mayo Clinic, Rochester, Minnesota, USA, mayo.edu; ^4^ Department of Urology, Mayo Clinic, Rochester, Minnesota, USA, mayo.edu; ^5^ Department of Internal Medicine, Division of Oncology, Mayo Clinic, Phoenix, Arizona, USA, mayo.edu; ^6^ Department of Radiology, Division of Nuclear Medicine, Mayo Clinic, Rochester, Minnesota, USA, mayo.edu

**Keywords:** lutetium-177-PSMA, PGCC, pleomorphic giant cell, pleomorphic giant cell carcinoma, Pluvicto, prostate cancer, radioligand therapy

## Abstract

Pleomorphic giant cell carcinoma (PGCC) of the prostate is an extremely rare and aggressive malignancy with limited treatment options. We present a case report of metastatic PGCC patients treated with ^177^Lu‐PSMA‐617 radioligand therapy (Pluvicto) after progression on standard therapies. Both patients had PSMA‐avid tumors on imaging. Molecular profiling revealed mutations in hallmark tumor suppressors including TP53, RB1, and PTEN. Both patients initially experienced partial radiographic response, in which one patient achieved stable disease for 9 months posttherapy. These findings suggest that PSMA‐radioligand therapy may be a reasonable treatment for PGCC patients, but underscore the need for additional therapeutic options.

## 1. Introduction

Pleomorphic giant cell carcinoma (PGCC) of the prostate is an exceedingly rare and aggressive variant of prostate cancer (PC) for which there is limited understanding and effective treatments [1]. Although PGCC is recognized by the World Health Organization as a rare histologic variant of prostate acinar adenocarcinoma [2], our current knowledge regarding PGCC remains sparse. This poses a unique challenge in the diagnosis and treatment of this aggressive form of PC.

PGCC tumors are frequently characterized by the presence of giant, anaplastic cells with pleomorphic nuclei on histologic examinations [3, 4]. The pleomorphic component of PGCC tumors ranges from 5% to 100% [5]. Although the degree of pleomorphism does not correlate with overall survival, patients with PGCC tumors are more likely to have acquired metastasis. It is possible that certain features of PGCC may portend a worse clinical prognosis. Indeed, recent studies have shown that overall survival was 3–60 months in 10 patient cases from the time of PGCC diagnosis [6]. Diagnosis of PGCC is challenging as enlarged, pleomorphic giant cells are not unique to PCs. Lung, pancreas, and bladder tumors are also known to share these morphologic features [7–9]. Therefore, it is essential to leverage orthogonal approaches including clinical history (such as known primary prostate adenocarcinoma), radiographic imaging, and biochemical and immunostaining with conventional prostate markers to diagnose this rare variant of PC.

Despite the advent of immunologic and chemotherapeutic agents, the treatment of metastatic castration‐resistant prostate cancer (CRPC) remains a challenge. Currently, a broad repertoire of treatment agents is available for the management of prostate cancer [10]. Androgen deprivation therapy (ADT) and androgen receptor pathway inhibitors (ARPI) along with chemotherapy are commonly effective in prostate adenocarcinoma. Yet, CRPC tumors are likely to acquire resistance in an androgen‐axis dependent or independent manner [11, 12]. There is growing evidence that histologic features of PGCC are more likely to be present in metastatic CRPC that has already received conventional PC therapies [1, 13, 14]. This raises an interesting question of whether metastatic CRPC tumors with PGCC histologic phenotypes may be more prone to develop resistance.

Given the sparse literature and treatment modalities for PGCC, there is a mounting need for promising adjuvant therapies for this aggressive subtype of PC. The recent VISION trial highlights the potential of harnessing lutetium‐based prostate‐specific membrane antigen (PSMA) radioligand therapy to treat metastatic CRPC [15]. PSMA is a Type II glycoprotein transmembrane protein that is highly expressed in metastatic CRPC compared with native host tissue [16, 17]. The ^177^Lu‐PSMA‐617 radioligand, which specifically targets PSMA, was found to prolong overall survival in conjunction with standard of care in patients with metastatic disease. Therefore, radioligand therapy may be effective for patients harboring PGCC tumors that highly express PSMA. To our knowledge, there has been no published literature on patients with PGCC tumors that received PSMA radioligand therapy. Here, we present clinical responses and molecular profiles of two cases of PSMA‐avid PGCC tumors exposed to 177Lu‐PSMA‐617 radioligand therapy.

## 2. Methods

### 2.1. Patient Samples

We identified two patients at a single center (Mayo Clinic Cancer Center) with PGCC who received lutetium‐based PSMA therapy, Pluvicto. Patients received Pluvicto 200 mCi every 6 weeks for a planned duration of six cycles. Patients were followed longitudinally, and clinical data were annotated by trained research personnel. Medical record review, patient samples, and analysis were approved by the Mayo Clinic Institutional Review Board.

The Prostate Cancer Working Group 3 (PCWG3) Criteria was used to define progression of CRPCs. Biochemical progression was defined as a 25% increase in PSA over nadir. Radiographic evidence of PC progression was evaluated using the RECIST 1.1 criteria, as described by the PC Clinical Trials Working Group 3 [18].

### 2.2. Sequencing Analysis and Immunochemistry

Targeted next‐generation sequencing (NGS) of liquid biopsies was performed and analyzed with Guardant 360 (G360, Guardant Health). This molecular platform detects somatic mutations in a panel of 74 cancer‐related genes and 90 pan‐cancer microsatellite sites. As an orthogonal approach, molecular data was collected and analyzed from metastatic and primary PGCC tumors using high‐throughput NGS screening that profiles a targeted panel of oncogenic variants (MayoComplete Solid Tumor Panel, xT panel from Tempus Labs, and Caris Life Sciences). Immunohistochemistry was performed at Mayo Pathology Core with standard H&E and PSMA stains.

## 3. Case 1 Presentation

### 3.1. Clinical History

**Figure 1:**
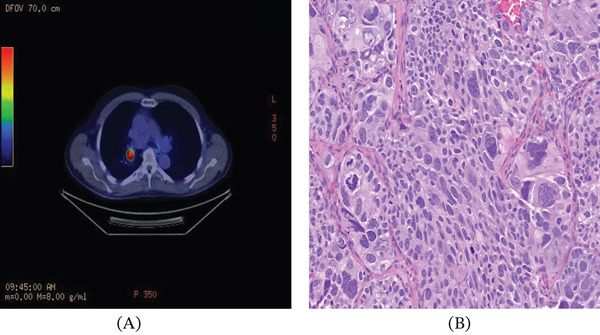
PSMA PET‐CT imaging and immunohistochemistry for Patient 1. (A) PSMA PET‐CT with avid uptake in the right hilar lymph node. (B) Metastatic lung lesion. Hematoxylin and eosin staining with irregular, giant cells and pleomorphic nuclei.

A 65‐year‐old male with a history of hyperlipidemia and a family history of PC presented to our outpatient primary care clinic for elevated PSA. On presentation, the patient was asymptomatic. PSA was measured at 5.6 ng/mL. MRI imaging had revealed an enlarged mass in the peripheral zone of the prostate with radiographic findings that were suspicious for malignancy. Subsequent bone scans did not show signs of metastatic disease. The patient underwent a radical prostatectomy with pelvic lymph node dissections, which led to undetectable PSA levels < 0.1 ng/mL. Histopathologic evaluation of biopsy samples was consistent with prostate adenocarcinoma (Gleason 4 + 5). His PSA remained low for 18 months before biochemical recurrence developed (PSA > 0.2 ng/mL) and was treated with androgen deprivation (leuprolide and bicalutamide) and radiation therapies. Following completion of his treatment course, his PSA remained undetectable for 14 months. Unfortunately, subsequent PET‐CT findings were concerning for metastatic disease with osseous and lymph node involvement. He was treated with salvage radiation treatment, ADT (bicalutamide and leuprolide), and combination carboplatin and docetaxel chemotherapies. Thereafter, the patient remained on ADT without radiographic evidence of progression for 11 months, until surveillance imaging detected new metastatic lesions in the right lung. Biopsy of one of these lesions revealed metastatic prostate adenocarcinoma with features of pleomorphic giant cells with bizarre nuclei, consistent with PGCC (Figure 1B). The patient was treated with thoracoscopy and wedge resection of the right lung, followed by ADT and surveillance imaging.

Approximately 10 months later, reassessment with PSMA PET‐CT scan showed PSMA‐avid lesions in the bilateral lungs and right hilar lymph node with miPSMA expression score of 3 (Figure 1A). Since the patient harbored PSMA‐positive tumors, he completed six cycles of Pluvicto. He tolerated the therapy with minimal fatigue and relative absence of other adverse events ascribed to this treatment [15], including nausea, vomiting, diarrhea, and abdominal pain. The patient did not experience biochemical progression and had achieved partial radiographic response for approximately 8 months after initiation of PSMA radioligand therapy. After Pluvicto treatment, the patient unfortunately experienced disease progression and was subsequently treated with three cycles of carboplatin and cabazitaxel and four cycles of PSMA actinium, which led to undetectable PSA and partial radiographic response. Later on, repeat surveillance scan showed disease progression with new and worsening liver and bone metastasis. The patient completed four cycles of combination of cisplatin with etoposide without effective clinical response. He was later transitioned to hospice care before passing. The patient survived approximately 9 years from the initial diagnosis of prostate adenocarcinoma and 4 years from transformation to PGCC.

### 3.2. Molecular Profiling

The patient underwent a transrectal ultrasound‐guided biopsy of the prostate, which confirmed prostate adenocarcinoma with Gleason scores 7 (3 + 4) and 6 (3 + 3) from the right and left prostate, respectively. In line with these findings, specimens from radical prostatectomy also showed Gleason score 9 (4 + 5).

Approximately 3.5 years from the initial diagnosis of his prostate adenocarcinoma, the primary tumor had undergone metastatic dissemination based on surveillance imaging. Tumor biopsy of the right lung nodule revealed metastatic PC with histologic features of PGCC, consisting of bizarre, giant cells with nuclear pleomorphism (Figure 1B). To investigate the genetic profile of the PGCC tumor, high‐throughput sequencing analysis was performed to detect cancer‐related somatic mutations in circulating cell‐free DNA (cfDNA). The PGCC tumors were found to harbor microsatellite stable genomes with deleterious mutations in *TP53* **(**K132 frameshift (fs), 0.2% cfDNA and R273C, 1.5% cfDNA) and *RB1* (L769*Δ* premature stop codon, 1.8% cfDNA) (Table 1). These results were recapitulated at an outside institution, in which NGS profiling of metastatic lung tissue detected pathogenic *TP53* (R273C, 69% variant allele frequency VAF), *RB1* (L769*Δ*, 78% VAF), and additional *KDM6A* (c1922_1923+7del9, 79% VAF) mutations. Tumor mutational burden remained low at 3 mutations/Mb. Interestingly, we observed loss of *TP53* R273C and *RB1* L769*Δ* alterations in cfDNA after completion of standard PC therapies, which also remained unchanged while on Pluvicto (Table 1).

**Table 1:** Molecular liquid biopsy profiling before and after standard PC treatment for Case 1.

	Before standard PC treatment	After standard PC treatment
Gene	Mutation (cfDNA %)	Mutation (cfDNA%)
*TP53*	K132fs (0.2%)	K132fs (0.3%)
	R273C (1.5%)	Undetectable
*RB1*	L769*Δ* (1.8%)	Undetectable

*Note:* The symbol *Δ* denotes premature stop codon; fs: frame shift mutation.

## 4. Case 2 Presentation

### 4.1. Clinical History

A 64‐year‐old male with a history of heart failure with preserved ejection fraction, atrial fibrillation, and obstructive sleep apnea had presented to Mayo Clinic from an outside institution with metastatic PGCC. He was evaluated at our emergency department for worsening constipation and urinary retention. Initial radiographic imaging demonstrated an enlarged prostate and elevated PSA at 55.1 ng/mL. Patient was referred to the Mayo Clinic for further evaluation after PET‐CT scan revealed diffuse bone metastasis. A transrectal ultrasound‐guided biopsy of the prostate confirmed the diagnosis of metastatic PGCC. In the absence of prior cancer history or anticancer treatments, it is unclear if the patient acquired PGCC de novo or secondary to transformation from primary prostate adenocarcinoma. Interestingly, the patient had a history of chronic occupational exposure to unknown chemicals in the 1980s and had a paternal uncle afflicted with PC.

**Figure 2:**
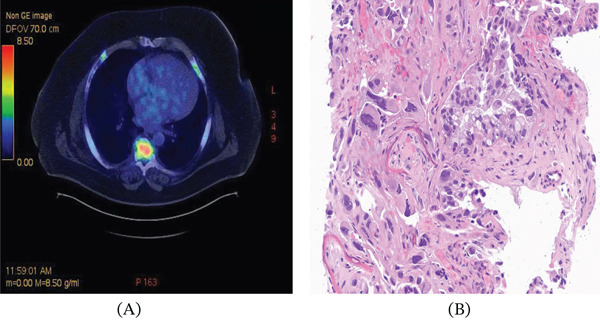
PSMA PET‐CT imaging and immunohistochemistry for Patient 2. (A) PSMA PET‐CT with avid uptake in thoracic vertebrae. (B) Metastatic bone lesion. Hematoxylin and eosin staining with irregular, giant cells and pleomorphic nuclei.

The patient was initiated on ADT and combination chemotherapy (docetaxel and carboplatin). PSA normalized to < 1 ng/mL. Four months after completion of treatment, the patient experienced progression of his disease. PSMA PET‐CT scans demonstrated innumerable PSMA‐avid lesions in the lymph nodes and bones with miPSMA expression score of 3 (Figure 2A). The patient did not achieve radiographic response on abiraterone. Considering the treatment‐refractory PGCC tumors and PSMA‐positive radiographic findings, the patient was initiated on Pluvicto. He completed four cycles of Pluvicto and subsequent palliative radiation therapy with some partial radiographic response. However, Pluvicto administration was discontinued due to clinical deterioration and eventual tumor progression. The patient eventually transitioned to hospice care before passing away. He survived for approximately 7 months after starting Pluvicto and 21 months after the initial PGCC diagnosis.

### 4.2. Molecular Profiling

The patient underwent a transrectal ultrasound‐guided biopsy of the prostate, which confirmed PGCC with Gleason scores 9 (5 + 4) and perineural invasion involvement. Tumor biopsy of metastatic bone lesions revealed PGCC tumors consisting of giant cells with nuclear pleomorphism (Figure 2B). To interrogate the cancer‐associated genomic landscape in PGCC tumors, we performed liquid‐biopsy genomic profiling of cfDNA. We observed enrichment of deleterious alterations in *TP53* (R249T, 18.6% cfDNA), *PTEN* (T319*Δ* premature stop codon, 9.9% cfDNA), and *KRAS* (Y166N 35.8% cfDNA) (Table 2). These mutations were subsequently confirmed through targeted NGS panel sequencing of malignant prostate tissue biopsies, which revealed *TP53* R249T, *PTEN* T319 deletion, *KRAS* Y166N, and additional *TMPRSS2-ERG* and *KLC-RAF1* fusion variants (Table 2). Tumor mutational burden remained low at 5.5 mutations/Mb with microsatellite stable genomes.

**Table 2:** Molecular liquid biopsy profiling before and after standard PC treatment for Case 2.

	Before standard PC treatment	After standard PC treatment
Gene	Mutation (cfDNA %)	Mutation (cfDNA%)
TP53	R249T (18.6%)	R249T (4.4%)
PTEN	T319*Δ* (9.9%)	T319*Δ* (2.1%)
KRAS	Y166N (35.8%)	Undetectable

*Note:* The symbol *Δ* denotes premature stop codon.

We also investigated alterations in mutations in response to anticancer treatments. After completion of standard PC therapies, the mutation frequency in cfDNA decreased *TP53* (18.6%–4.4%) and *PTEN* (9.9%–2.1%). Remarkably, the *KRAS* Y166N mutation was undetectable. As an orthogonal approach, NGS analysis on biopsied metastatic lesions revealed genomic aberrations in *TP53* (R249T, 23.0% VAF), *PTEN* (T139*Δ*, VAF 23.2%), *TMPRSS2-ERG*, and an additional *MAP3K1* copy number loss. Tumor mutational burden increased from 5.5 to 7.4 mutations/Mb with microsatellite‐stable genomes.

## 5. Conclusion

PGCC of the prostate is an exceptionally rare subtype of prostate adenocarcinoma. This malignant disease is defined by the histologic presence of bizarre, giant cells with pleomorphic nuclei [1, 3–5]. Despite the paucity of literature on PGCC, there is growing evidence that tumors with such morphologic features are more likely to be linked with therapeutic resistance, disseminating metastases, and poor patient survival [1, 5, 6, 13, 14]. It remains unknown whether PGCC is present de novo in early primary PCs or acquired through malignant transformation during tumor progression. Although beyond the scope of this study, further work is required to refine our understanding of the underlying biology and genetics that drive PGCC formation.

There are limited studies that address the management of patients with PGCC who have exhausted conventional treatment options. In 2022, Pluvicto became the first radioligand therapy that was approved by the FDA for the treatment of PSMA‐positive, metastatic CRPC [19]. The VISION trial has shown that PSMA‐radioligand therapy may provide clinical benefit for patients with advanced CRPC [15]. Recently, the PSMAfore Phase III trial has also demonstrated that PSMA‐radioligand therapy improved radiographic progression‐free survival by nearly 59% in their primary analysis compared with patients treated with ARPI (HR: 0.41, 95% CI: 0.29–0.56, *p* < 0.000) [20]. Beyond the US, the implementation of PSMA‐radioligand therapy for metastatic CRPC has also risen over the years as another therapeutic option worldwide, including Europe [21, 22]. In 2025, the FDA has expanded the approval for Pluvicto to include patients with PSMA‐positive, metastatic CRPC who have trialed ARPI and are candidates to defer chemotherapy [23]. Therefore, careful design of radioligands may serve as promising avenues for targeting PSMA‐expressing tumors. To our knowledge, there is no published data on the clinical response to PSMA radioligand therapy in patients with PGCC.

We present two cases involving patients with treatment‐refractory PGCC tumors who underwent Pluvicto radioligand therapy. Both patients exhibited PSMA‐positive tumors on imaging. The median age of PGCC diagnosis is 71 years with Gleason scores above 9 [1, 3, 5]. This is congruent with our patients who were 65 and 64 years old, both harboring PGGC tumors with Gleason scores of 9. Following cancer relapse to standard treatments, both patients were selected to receive Pluvicto after confirmation of PSMA‐avid lesions on imaging. Initially, both patients were able to achieve radiographic and biochemical stable disease. The first patient has completed the full six cycles of Pluvicto and achieved partial radiographic response without biochemical recurrence for approximately 8 months postinitiation of radioligand therapy. On the other hand, Pluvicto was discontinued after Cycle 4 for the second patient due to cancer progression. He survived 7 months from the start of Pluvicto therapy.

The genomic landscape of metastatic PC is highly complex and heterogeneous, where diverse molecular alterations can influence therapeutic response, including clinical outcomes to PSMA‐targeted radioligand therapies [24–27]. Moreover, this study reveals the complex mutational heterogeneity of PGCC tumors. Pathogenic TP53 alterations were detected in cancer cells derived from both patients. PGCC tumors in the first patient also harbored RB1 mutations, which aligns with prior studies that show frequent loss of TP53 and RB1 in PGCC [1, 5, 28]. In contrast, PGCC tumors from the second patient had acquired additional mutations, which include PTEN, TMPRSS2‐ERG, MAP3K1, and KRAS somatic variants. Overall, these somatic mutations abrogate key oncogenic pathways that are recurrent in PC [1, 29–32].

We are limited in the number of PGCC patient cases who received radioligand therapy. Yet, the survival outcomes observed in our patients may reflect underlying biological differences in their tumors. The first patient had undergone standard treatments for primary prostate adenocarcinoma, which later transformed into metastatic PGCC that was responsive to Pluvicto. In contrast, the second patient was initially diagnosed with metastatic PGCC before undergoing standard treatments and subsequent radioligand therapy. Therefore, it is possible that the second patient experienced a less favorable clinical response to Pluvicto due to higher tumor burden and more aggressive cancer progression. The metastatic PGCC tumors may have already acquired additional pathogenic mutations, fostering the emergence of subclonal populations that are highly resistant to radioligand therapy. In support of this hypothesis, PGCC tumors in the second patient exhibit an increased tumor mutational burden with several additional mutations in PTEN, TMPRSS2‐ERG, KRAS, and MAP3K1 compared with the first patient.

Interestingly, therapeutic challenges with conventional PC agents may induce negative selection of distinct genetic alterations. Standard treatments led to loss of TP53 and RB1 mutations in cfDNA from the first patient. Likewise, TP53, PTEN, and KRAS variants decreased in cfDNA after treatment in the second patient. These findings are incongruent with previous work that highlights selective enrichment of TP53 and PTEN mutations in metastatic CRPC compared with their primary cancer counterparts [33, 34]. In contrast to prior associations of RB1 and KRAS mutations with late‐stage PCs [35–37], we observed loss of these mutations in PGCC. Taken together, these results raise the possibility that anti‐PC agents may enforce distinct selective pressures in PGCC compared with conventional CRPC, which may contribute to the tumor heterogeneity across different subtypes of PCs. However, it is important to acknowledge the limitations of high throughput sequencing of cfDNA, which has a clinical sensitivity and specificity of 85% and 99.6%, respectively, in comparison to tissue‐based NGS [38]. Therefore, comprehensive NGS of cfDNA may not recapitulate the full mutational landscape of PGCC as observed with whole‐genome sequencing on solid tumors.

A comprehensive understanding of the underlying molecular pathways and genetic landscape of PGCC will improve our ability to develop novel therapeutic agents and clinical guidelines for this unique variant of prostate adenocarcinoma. We presented two PGCC cases, which were initially responsive to Pluvicto. Despite the rarity of this disease, further work is required to define the molecular context in which Pluvicto will confer optimal clinical benefit for patients with PGCC. Careful selection of patients will also be required to identify the subset of patients who will likely achieve robust clinical response to treatment. Overall, this study opens a promising opportunity for radioligand therapy in the clinical management of patients with PGCC who exhausted conventional treatments.

**Nomenclature**
Pleomorphic giant cell carcinoma: (PGCC)
Castration‐resistant prostate cancer: (CRPC)
Prostate‐specific membrane antigen: (PSMA)
Androgen deprivation therapy: (ADT)
Androgen receptor pathway inhibitors: (ARPI)

## Author Contributions

**John Y. Kwon:** writing – original draft, writing – review and editing, visualization, conceptualization, investigation. **John C. Cheville:** writing – review and editing, visualization, investigation. **Miguel Muniz-Rincon:** writing – review and editing. **Mohamed E. Ahmed:** writing – review and editing. **Eugene D. Kwon**: writing – review and editing. **Irbaz Riaz:** writing – review and editing. **Dan S. Childs:** writing – review and editing. **Geoffrey Johnson:** writing – review and editing. **Oliver Sartor:** writing – review and editing. **Lance C. Pagliaro:** writing – review and editing. **Jacob J. Orme:** writing – original draft, writing – review and editing, visualization, investigation, conceptualization, supervision.

## Funding

No funding was received for this manuscript.

## Disclosure

All authors have read and approved the final version of the manuscript. Corresponding author, Dr. Jacob Orme had full access to all of the data in this study and takes complete responsibility for the integrity of the data and the accuracy of the data analysis.

## Consent

Written informed consents were obtained from both patients for their anonymized information to be published in this article. No patient‐identifiable data was included in this case report. The study was approved by the Mayo Clinic Institutional Review Board (22‐010856).

## Conflicts of Interest

The authors declare no conflicts of interest.

## Data Availability

The data that support the findings of this study are available from the corresponding author upon reasonable request.
